# Associations of ultrasound estimated early mid pregnancy visceral and subcutaneous fat depths and early pregnancy BMI with adverse neonatal outcomes

**DOI:** 10.1038/s41598-021-84045-8

**Published:** 2021-02-25

**Authors:** Emelie Lindberger, Anna-Karin Wikström, Eva Bergman, Karin Eurenius, Ajlana Mulic-Lutvica, Linda Lindström, Inger Sundström Poromaa, Fredrik Ahlsson

**Affiliations:** Department of Women’s and Children’s Health, Uppsala University, 751 85 Uppsala, Sweden

**Keywords:** Epidemiology, Paediatric research

## Abstract

This study investigated whether maternal central adiposity and body mass index (BMI) were associated with neonatal hypoglycemia and adverse neonatal outcomes. A cohort study was performed at Uppsala University Hospital, Sweden, between 2015 and 2018. Visceral and subcutaneous fat depths were measured by ultrasound at the early second-trimester anomaly scan in 2771 women giving birth to singleton infants. Body mass index was assessed in early pregnancy. Logistic regression models were performed. Adjustments were made for age, BMI (not in model with BMI as exposure), smoking, maternal country of birth, and parity. Outcomes were neonatal hypoglycemia (blood glucose concentration < 2.6 mmol/l), a composite of adverse neonatal outcomes (Apgar < 7 at 5 min of age, or umbilical artery pH ≤ 7.0, or admission to neonatal intensive care unit), and the components of the composite outcome. Visceral and subcutaneous fat depths measured by ultrasound in early mid pregnancy were not associated with any of the outcomes in adjusted analyses. For every unit increase in BMI, the likelihood of neonatal hypoglycemia increased by 5% (aOR 1.05, 95% CI 1.01–1.10), the composite outcome by 5% (aOR 1.05, 95% CI 1.01–1.08), and admission to neonatal intensive care unit by 6% (aOR 1.06, 95% CI 1.02–1.10).

## Introduction

The prevalence of overweight and obesity is increasing worldwide, affecting more than 1.9 billion people^1^. In addition, a large proportion of the fertile population is afflicted by these conditions; approximately 20% of expecting mothers are overweight and 10% are obese in early pregnancy^2^.

It is well established that overweight and obesity during pregnancy are predisposing factors for several adverse perinatal outcomes, for both the mother and the infant^3–6^. Neonatal complications include increased birthweight, respiratory distress, hypoglycemia, and admission to neonatal intensive care unit (NICU)^7^.

In non-pregnant individuals, a body fat distribution characterized by central adiposity increases the risk of obesity-related complications^8–10^. Central adiposity is often accompanied by insulin resistance, hyperlipidemia, and low-grade inflammation^11,12^, which are suggested as the underlying mechanisms of obesity-related complications^13^. Central adiposity can be further subdivided into abdominal subcutaneous fat and intra-abdominal visceral fat^14^. Especially accumulation of intra-abdominal visceral fat is associated with type 2 diabetes and cardiovascular disease^15^, and is therefore considered unhealthier in a metabolic sense compared with a body distribution characterized by subcutaneous fat accumulation in the gluteofemoral region^16^.

Today, body mass index (BMI) is used in risk stratification of pregnant women. A disadvantage of BMI is that it does not take into account body fat distribution. Hence, clinicians lack an effective way to assess individual risk among overweight pregnant women, and a more precise risk marker of metabolic risk is needed.

We have previously found associations between visceral fat depth, a measure of central adiposity, in early mid pregnancy and infant birthweight, and the likelihood of giving birth to an infant large for gestational age, independent of early pregnancy BMI^17^. These findings are in line with the results of several previous studies^18–24^ and suggest a relation between maternal fat distribution and fetal development. Supposedly, maternal central adiposity could be associated with metabolic processes in the fetus, making the newborn infant more susceptible to neonatal compromise such as hypoglycemia and other perinatal complications.

Whether body fat distribution in the pregnant woman affects the risk of hypoglycemia and adverse neonatal outcomes is scarcely studied. To our knowledge, the relation between maternal central adiposity and neonatal hypoglycemia is evaluated by one previous study^25^, and adverse neonatal outcomes by two^25,26^. These studies report associations between maternal central adiposity and the outcomes. However, only abdominal subcutaneous fat tissue was used as a proxy for central adiposity. None of the studies assessed the visceral fat compartment, which is more strongly associated with complications of overweight and obesity in non-pregnant individuals^11^.

We hypothesize that infants of mothers with central adiposity and especially visceral fat accumulation have an increased likelihood of a dysfunctional postnatal metabolic adaption and increased likelihood of neonatal hypoglycemia, independent of early pregnancy BMI. Furthermore, we hypothesize that infants of mothers with visceral fat accumulation run increased risk of adverse neonatal outcomes.

In this population-based study including 2771 pregnancies, we investigated the associations of ultrasound estimated maternal early mid pregnancy visceral fat depth, subcutaneous fat depth, and early pregnancy BMI with neonatal hypoglycemia and adverse neonatal outcomes, defined as low Apgar score, acidosis at delivery, and admission to NICU.

## Material and methods

This cohort study was performed at Uppsala University Hospital (Uppsala, Sweden) between January 2015 and April 2018. Ethical approval was obtained to implement a new clinical routine, visceral fat depth (VF) and subcutaneous fat depth (SCF) measurements, and to evaluate this routine by linkage to standardized hospital electronic medical records on maternal, obstetric, and perinatal health care. Following linkage, the study population database was anonymized. The study was approved by the Regional Ethical Review Board in Uppsala on September 24th 2014 (Dnr:2014/353). All research was performed in accordance with relevant national and international guidelines. Informed consent was waived by the Swedish Ethical Review Authority (Dnr: 2019-00391). This was not required as this study was register-based with anonymized data. In general, large registry-based studies in Sweden do not require informed consent. It is explained by Ludvigsson et al.^27^ that as long as a registry-based study is deemed ethical by the ethical committee, it is assumed that the participants do not object to the research.

Eligible study participants were women attending the second-trimester anomaly scan at Uppsala University Hospital from January 2015 to December 2017 (n = 12,744). Out of these, 2844 had their scan performed by a midwife trained in fat depth measurements. It was a matter of coincidence whether the pregnant woman was examined by a midwife trained in fat depth measurements, since the personnel booking the ultrasonography appointments were not aware of this study. Out of the 2844 women, 94 women were excluded from further analysis due to missing information on BMI at first antenatal visit (n = 49), inaccessible electronic medical records (n = 1), multiple pregnancy (n = 7), intrauterine fetal death (n = 5), missing data from the delivery (n = 1) and missing VF or SCF measures (n = 10). The final study population consisted of 2771 women who gave birth to singleton infants between June 2015 and April 2018.

In addition, a sensitivity analysis was performed where the population was restricted to healthy women. We excluded 63 women due to chronic illness (diabetes mellitus type 1 or type 2, rheumatic disease, epilepsy, inflammatory disease, or essential hypertension), 34 due to gestational diabetes, 83 due to gestational hypertension, and 95 due to preeclampsia. In total, the healthy subgroup consisted of 2496 women and child dyads.

### Data collection

The method of fat depth measurements, performed as per Armellini et al.^28^ with a minor modification regarding the placement of the ultrasound probe, is described in detail elsewhere^17^. The following information was extracted from the women’s standardized antenatal electronic medical records: BMI (kg/m^2^), age (years), smoking status at first antenatal visit (yes or no), parity (nulliparous or parous), and maternal country of birth (EU or outside EU). At first antenatal visit, the midwife recorded information on chronic illnesses using checkboxes in the standardized antenatal electronic medical record. The information was supplemented with obstetric diagnoses by the obstetrician at discharge from the delivery unit. We extracted data from the women’s standardized antenatal electronic medical records on the following diagnoses according to the International Classification of Diseases 10 (ICD-10): diabetes mellitus type 1 and type 2 (E10, E11), rheumatic disease (L40, M05, M32, M35, M45), epilepsy (G40), inflammatory disease (D69, K50, K51, K90), essential hypertension (I10), gestational diabetes (O244), gestational hypertension (O13), and preeclampsia (O14). Gestational diabetes was defined as fasting plasma glucose ≥ 7.0 mmol/l or plasma glucose ≥ 9.0 mmol/l 2 h after oral intake of 75 g glucose. At Uppsala University Hospital, only women identified as having an increased risk for gestational diabetes undergo oral glucose tolerance tests.

Data on gestational age, sex, birthweight, umbilical artery pH, Apgar score, and blood glucose concentrations were extracted from the standardized pediatric electronic medical records and added to the study population database. Data on admission to NICU were obtained from the Swedish Neonatal Quality Register (SNQ). Term birth was defined as delivery between 37 ^+ 0^ and 41 ^+ 6^ weeks of gestation. We defined an episode of hypoglycemia as a blood glucose concentration < 2.6 mmol/l. Since many infants have a physiological nadir in blood glucose concentration between one to two hours after birth^29^, we only investigated hypoglycemic episodes after the age of two hours.

Due to a very large number of blood glucose measures, we extracted one blood glucose concentration per infant (if available) for the following time intervals: 60 min intervals from age 2–12 h, 120 min intervals from age 12–48 h, and 360 min intervals thereafter. If more than one blood glucose value was available in the specified time interval, the following priority for extraction was applied: firstly, blood sample analyzed on a blood gas analyzer (ABL800 Radiometer) at the ward, and secondly, by Abbott Architect c16000 at the hospital laboratory. If two or more blood glucose measures in a specified time interval were analyzed by the same method, the lowest blood glucose concentration was chosen.

Blood samples for blood glucose measurement were taken according to clinical praxis. At Uppsala University Hospital, infants identified as being at risk for neonatal hypoglycemia undergo regularly blood glucose monitoring according to screening protocols. At risk categories include being preterm (< 36 ^+ 0^ weeks gestation), small for gestational age (SGA) (birthweight below minus two standard deviation scores of the mean birthweight for the gestational age^30^), having a diabetic mother receiving pharmacological treatment for her diabetes, and being identified as at risk by the medical team (for example due to feeding problems or jitteriness). The neonatal hypoglycemia screening protocol for preterm infants and infants born SGA implies a first blood glucose measurement prior to the second feeding (at approximately age three hours) and thereafter prior to every second feeding. If the blood glucose measures are normal (≥ 2.6 mmol/l), the test frequency is reduced, and the screening ended after 48 h. The screening protocol for infants of diabetic mothers with pharmacological diabetes treatment implies a blood glucose measurement prior to the second feeding (at approximately age 3 h), and thereafter prior to every feeding. The test frequency is reduced to every other feeding after two consecutive normal measures (≥ 2.6 mmol/l), and the screening is ended after 14 h. The protocol for infants with hypoglycemia (< 2.6 mmol/l) implies blood glucose measurement prior to every feeding. After two consecutive normal measures (≥ 2.6 mmol/l), the test frequency is reduced. Infants of diabetic mothers with dietary treatment undergo blood glucose measurement at age three hours and six hours. If the blood glucose measures are normal (≥ 2.6 mmol/l), no further testing is done.

### Outcomes

Two primary outcomes were evaluated (1) neonatal hypoglycemia, defined as a blood glucose measure < 2.6 mmol/l between the age of 2 h and 7 days, and (2) a composite of adverse neonatal outcomes (Apgar < 7 at 5 min of age, or umbilical artery pH ≤ 7.0, or admission to NICU). Secondary outcomes included the components of the composite outcome.

### Statistical analyses

Sample size tables for logistic regression^31^ were used to determine the required sample number. The outcome prevalence was estimated to be 10%. For simple logistic regression with α = 5%, a sample size of 2236 would result in a power of 80% to detect an OR 1.20 at one standard deviation above the mean of the exposure. Normal distribution of VF, SCF, and early pregnancy BMI measures were assessed in the cohort. Pearson’s correlation coefficients were calculated to evaluate the relations between VF, SCF, early pregnancy BMI, and maternal age. T-tests were used to compare VF, SCF, and early pregnancy BMI between groups defined by clinical and demographic parameters. Chi square tests were used to assess relations between categorical covariates and outcomes. When few observations were expected (< 5), Fisher’s exact test was performed.

Simple and multiple logistic regression analyses were performed to separately examine the association between VF (in 5 mm intervals), SCF (in 5 mm intervals), and BMI (kg/m^2^) and the likelihood of neonatal hypoglycemia. Subsequently, simple and multiple logistic regression models were performed to evaluate the likelihood of the composite outcome, and the components of the composite outcome (Apgar < 7 at 5 min of age, umbilical artery pH ≤ 7.0, and admission to NICU). In the models with VF and SCF as exposures, adjustments were made for maternal age, early pregnancy BMI, smoking status at first antenatal visit, maternal country of birth, and parity. In the model with early pregnancy BMI as exposure, the same adjustments were made except for BMI. Directed acyclic graphs were used to select covariates. Variables included in the directed acyclic graphs were either known to be associated with the exposures and outcomes, or considered clinical relevant.

We imputated information on smoking status at first antenatal visit, since this information was missing in 51% of the women. The imputation was performed by the random hot deck method^32^. By using information on maternal age, BMI, and country of birth, controls for women with known smoking status were matched among women with unknown smoking status. Thereafter, a random control was drawn for every woman with known smoking status, and given the same smoking status as its match. After the imputation, the prevalence of smoking at the first antenatal visit in the cohort was 3.6%.

Data were missing on Apgar score and umbilical artery pH on some of the infants (Supplementary Table 1). We analyzed the cases that had available data, hence, the number of mother–child dyads differed between the different outcomes studied. We did not perform any data imputation besides that on smoking status at first antenatal visit.

**Table 1:** Descriptive characteristics of the study population.

	Variable	Cohort
**Women**	N	2771
	Age, years (mean, range)	30.3 (16‒45)
	Nulliparous, n (%)	1185 (42.8)
	Early pregnancy BMI kg/m^2^ (mean ± SD)	25.1 ± 5.0
	BMI < 18.5 kg/m^2^ (underweight), n (%)	62 (2.2)
	BMI 18.5–24.9 kg/m^2^ (normal weight), n (%)	1568 (56.6)
	BMI 25.0–29.9 kg/m^2^ (overweight), n (%)	703 (25.4)
	BMI ≥ 30.0 kg/m^2^ (obesity), n (%)	438 (15.8)
	Smoking at first antenatal visit, n (%)	99 (3.6)
	Country of birth within the EU, n (%)	2399 (86.6)
	Gestational diabetes mellitus, n (%)	35 (1.3)
**Offspring**	N	2771
	Gestational length, days (mean ± SD)	278 ± 12
	Preterm, n (%)	118 (4.3)
	Post-term, n (%)	150 (5.4)
	Birthweight, g (mean ± SD)	3576 ± 530
	Small for gestational age, n (%)	26 (0.9)
	Large for gestational age, n (%)	134 (4.9)

*BMI* body mass index, preterm, < 37 ^+ 0^ weeks of gestation; post-term, > 41 ^+ 6^ weeks of gestation; small for gestational age, birthweight below minus two standard deviation scores of the mean birthweight for the gestational age and sex^30^; large for gestational age, birthweight above plus two standard deviation scores of the mean birthweight for the gestational age and sex^30^.

IBM SPSS Statistics version 27 was used for all statistical analyses. Statistical significance was considered to be indicated by a nominal two-side *P*-value < 0.05.

## Results

### Maternal and infant characteristics

The women had a mean age of 30.3 years (range 16–45 years), 1185 (42.8%) were nulliparous, and 1141 (41.2%) were either overweight or obese. The mean early pregnancy BMI was 25.1 kg/m^2^. The VF and SCF measurements were performed at mean 132 days of gestation (range 102‒188 days, standard deviation (SD) 6 days). There were no correlations between the gestational age at the ultrasound examination and the fat depths (Pearson’s r = 0.17 (VF) and r = 0.06 (SCF)). The prevalence of gestational diabetes was 1.3%. The mean gestational age at birth was 278 days (SD 12 days) and the mean birthweight was 3576 g (SD 530 g). The baseline characteristics are described in Table 1.

Out of the 2771 infants, 64 (2.3%) had an episode of neonatal hypoglycemia. Six (9.4%) of the infants suffering from hypoglycemia had a mother with gestational diabetes, eight (12.5%) were born SGA, and one (1.6%) was born preterm.

Data on the composite outcome (Apgar < 7 at 5 min of age, or umbilical artery pH ≤ 7.0, or admission to NICU), were complete in 2077 infants, and out of these, 137 (6.6%) had the composite outcome (Supplementary Table 1). Out of 2635 infants with available data on Apgar score, 42 (1.6%) had Apgar < 7 at 5 min of age (Supplementary Table 1). Data on umbilical artery pH were available on 2118 infants, and out of these, 19 infants (0.9%) had umbilical artery pH ≤ 7.0 (Supplementary Table 1). Ninety-nine of 2771 infants (3.6%) were admitted to NICU (Supplementary Table 1).

### VF, SCF and early pregnancy BMI in relation to covariates and to outcomes

Visceral fat depth ranged from 3 to 116 mm and SCF from 1 to 52 mm. Early pregnancy BMI correlated with VF and SCF, r = 0.49 and r = 0.67, respectively (Supplementary table 2).

**Table 2:** Comparison of visceral fat depth, subcutaneous fat depth, and early pregnancy BMI between groups defined by demographic and clinical parameters.

			Visceral fat depth (mm)		Subcutaneous fat depth (mm)		BMI (kg/m^2^)	
Demographic variables	n	%	Mean (± SD)	*P*	Mean (± SD)	*P*	Mean (± SD)	*P*
**Smoking status at first antenatal visit**^**a**^								
No	2672	3.6	44.4 (16.3)	0.433	**17.1 (7.6)**	**0.011**	**25.1 (5.0)**	**0.045**
Yes	99	96.4	45.7 (16.0)		**19.1 (8.9)**		**26.4 (6.5)**	
**Maternal country of birth**								
EU	2399	86.6	**43.9 (16.2)**	** < 0.001**	17.2 (7.7)	0.795	25.2 (5.0)	0.382
Outside EU	372	13.4	**47.9 (16.1)**		17.1 (7.2)		24.9 (5.0)	
**Parity**								
Nulliparous	1185	42.8	**43.4 (15.6)**	**0.002**	**18.2 (7.6)**	** < 0.001**	**24.8 (4.9)**	** < 0.001**
Parous	1586	57.2	**45.2 (16.7)**		**16.5 (7.6)**		**25.4 (5.1)**	

Data were analyzed using t-tests. Significant results are in bold.

^a^After imputation of data on smoking status.

BMI, body mass index.

Women who were smokers had higher SCF and higher early pregnancy BMI compared with women who did not smoke (Table 2). Women born outside EU had higher VF compared with women born in EU (Table 2). Parous women had higher VF, higher early pregnancy BMI, and lower SCF compared with nulliparous women (Table 2).

Mothers whose infants had hypoglycemia had higher SCF and higher BMI compared with those whose infants did not have hypoglycemia (Table 3). Women whose infants had the composite outcome or were admitted to NICU had higher SCF and higher early pregnancy BMI compared with mothers whose infants did not have these outcomes (Table 3).

**Table 3:** Comparison of visceral fat depth, subcutaneous fat depth, and early pregnancy BMI between groups defined by neonatal outcomes.

			Visceral fat depth (mm)		Subcutaneous fat depth (mm)		BMI (kg/m^2^)	
Neonatal outcomes	N	%	Mean (± SD)	*P*	Mean (± SD)	*P*	Mean (± SD)	*P*
**Hypoglycemia**								
Yes	64	2.3	46.9 (18.2)	0.213	**19.6 (8.0)**	**0.010**	**26.5 (5.1)**	**0.032**
No	2707	97.7	44.4 (16.2)		**17.1 (7.7)**		**25.1 (5.0)**	
**Composite outcome**^**a**^								
Yes	137	6.6	45.7 (16.8)	0.576	**18.7 (8.2)**	**0.042**	**26.3 (5.4)**	**0.006**
No	1940	93.4	44.9 (16.6)		**17.3 (7.8)**		**25.1 (5.1)**	
**Apgar < 7 at 5 min of age**								
Yes	42	1.6	44.5 (15.0)	0.888	17.4 (7.7)	0.939	25.5 (5.3)	0.651
No	2593	98.4	44.8 (16.3)		17.3 (7.7)		25.2 (5.1)	
**Umbilical artery pH ≤ 7.0**								
Yes	19	0.9	42.0 (16.7)	0.483	15.6 (6.3)	0.483	25.1 (3.5)	0.937
No	2099	99.1	44.7 (16.6)		17.3 (7.8)		25.2 (5.1)	
**Admission to NICU**								
Yes	99	3.6	46.8 (16.7)	0.139	**19.8 (8.4)**	**0.001**	**26.7 (5.7)**	**0.007**
No	2672	96.4	44.3 (16.2)		**17.1 (7.6)**		**25.1 (5.0)**	

Data were analyzed using t-tests. Significant results are in bold.

*NICU* neonatal intensive care unit, *BMI* body mass index.

^a^Apgar < 7 at 5 min of age, or umbilical artery pH ≤ 7.0, or admission to NICU.

### Infant outcomes in relation to covariates

The rate of neonatal hypoglycemia did not differ between nulliparous and parous women, between mothers who were smokers at first antenatal visit and those who were not, or between mothers born in EU and mothers born outside EU (Supplementary table 3).

Mothers of infants who had an Apgar score < 7 at 5 min of age were older compared with mothers whose infants did not (Supplementary Table 4). The rate of the composite outcome and the components of the composite outcome did not differ between nulliparous and parous women, between mothers who were smokers at first antenatal visit and those who were not, or between mothers born in EU and mothers born outside EU (Supplementary table 3).

**Table 4:** Associations between early mid pregnancy visceral fat depth, subcutaneous fat depth, early pregnancy BMI, and outcomes.

Outcome	Visceral fat depth						Subcutaneous fat depth						BMI					
	Unadjusted model			Adjusted model^b^			Unadjusted model			Adjusted model^b^			Unadjusted model			Adjusted model^c^		
	OR	CI	*P*	OR	CI	*P*	OR	CI	*P*	OR	CI	*P*	OR	CI	*P*	OR	CI	*P*
Neonatal hypoglycemia	1.05	0.97–1.13	0.215	1.01	0.93–1.10	0.849	**1.20**	**1.04–1.38**	**0.013**	1.11	0.91–1.36	0.307	**1.05**	**1.00–1.09**	**0.033**	**1.05**	**1.01–1.10**	**0.024**
Composite outcome^a^	1.01	0.96–1.07	0.618	0.97	0.91–1.03	0.296	1.10	1.00– 1.23	0.061	1.00	0.86–1.15	0.942	**1.04**	**1.01–1.08**	**0.007**	**1.05**	**1.01–1.08**	**0.005**
Apgar < 7 at 5 min of age	0.99	0.90–1.09	0.835	0.96	0.86–1.07	0.460	1.03	0.85–1.24	0.798	0.95	0.73- 1.24	0.717	1.01	0.96–1.07	0.651	1.02	0.96–1.08	0.571
Umbilical artery pH ≤ 7.0	0.95	0.83–1.10	0.499	0.95	0.81- 1.12	0.553	0.83	0.60–1.15	0.260	0.71	0.46–1.11	0.129	1.00	0.91–1.09	0.937	1.00	0.91–1.09	0.959
Admission to NICU	1.05	0.99–1.11	0.146	1.00	0.93–1.07	0.911	**1.22**	**1.08–1.36**	**0.001**	1.14	0.97–1.34	0.125	**1.06**	**1.02–1.09**	**0.002**	**1.06**	**1.02–1.10**	**0.001**

Data are odds ratios (OR) (95% confidence interval (CI)) for the change in outcome per 5 mm increase in fat depth and per unit increase in BMI (kg/m^2^).

Data were analyzed using logistic regression models. Significant results are in bold.

*NICU* neonatal intensive care unit, *BMI* body mass index.

^a^Apgar < 7 at 5 min of age, or umbilical artery pH ≤ 7.0, or admission to NICU.

^b^Adjustments in the model: early pregnancy BMI, age, smoking at first antenatal visit, parity, and country of birth.

^c^Adjustments in the model: age, smoking at first antenatal visit, parity, and country of birth.

### Logistic regression analyses on the association of early mid pregnancy VF, SCF, and early pregnancy BMI with infant outcomes

There was no association between VF and neonatal hypoglycemia (Table 4). A 5 mm increase in SCF was associated with a 20% increase in the odds of hypoglycemia (Odds ratio (OR) 1.20, 95% confidence interval (CI) 1.04–1.38), but this association was no longer significant after adjustments for covariates (Table 4). For every unit increase in early pregnancy BMI, the odds of neonatal hypoglycemia increased by 5% (OR 1.05, 95% CI 1.00–1.09) (Table 4). After adjustment for covariates, the result remained unchanged (Table 4).

Visceral fat depth and SCF were not associated with the composite outcome (Table 4). An increase in early pregnancy BMI by one unit was associated with a 5% increase in the odds of the composite outcome (OR 1.05, 95% CI 1.01 − 1.08) after adjustments (Table 4).

Visceral fat was not associated with the components of the composite outcome (Table 4). For every 5 mm increase in SCF, there was a 22% increase in the odds of admission to NICU (OR 1.22, 95% CI 10.8–1.36), but the association disappeared after adjustments (Table 4). One unit increase in early pregnancy BMI was associated with odds of admission to NICU that were 6% higher (OR 1.06, CI 1.02 − 1.09) (Table 4). The association remained after adjustments (OR 1.06, CI 1.02 − 1.10) (Table 4).

In the sensitivity analysis on a healthy subgroup of women (n = 2496), SCF and early pregnancy BMI were associated with neonatal hypoglycemia (OR 1.27, CI 1.01–1.60 and OR 1.08, CI 1.02–1.14, respectively) after adjustments (Supplementary table 5). The fat depths were not associated with the composite outcome or the components of it after adjustments (Supplementary table 5).

## Discussion

The results of this study demonstrated independent associations between early pregnancy BMI and neonatal hypoglycemia and between early pregnancy BMI and a composite of adverse neonatal outcomes (Apgar < 7 at 5 min of age, or umbilical artery pH ≤ 7.0, or admission to NICU). Additionally, early pregnancy BMI was associated with an increased likelihood of admission to NICU.

Visceral fat depth and SCF were not independently associated with the neonatal outcomes evaluated by this study. Contrary to our hypotheses, infants of mothers with central adiposity and specifically visceral fat accumulation did not have increased likelihood of neonatal hypoglycemia, or adverse neonatal outcomes.

Our findings confirm previous studies reporting associations between maternal BMI and neonatal hypoglycemia^6^, and between maternal BMI and adverse neonatal outcomes^3–5,33^. Factors that could contribute to the development of neonatal hypoglycemia in infants born to overweight and obese mothers include insulin resistance and high blood glucose levels in pregnant women with high fat mass^34^, and insulin resistance in fetuses of obese mothers^35^. Fetuses exposed to high amounts of glucose in utero adapt to this hyperglycemic state and produce higher amounts of insulin^36^. At birth, when the umbilical cord is cut, the continuous supply of glucose is disrupted. The infant must now undergo a metabolic adaption in order to adjust to intermittent feeding and fasting^37^. However, the slightly increased insulin levels make the infant more susceptible to hypoglycemia. The causal pathways behind the association between BMI and other adverse neonatal outcomes are not fully clarified. Vasudevan et al.^7^ suggest differences in the antenatal health surveillance between mothers with overweight and obesity versus normal weight mothers as one possible explanation. Furthermore, they propose that the standard assessments for fetal growth and health (fundal height and fetal movements) could be less reliable in mothers with overweight and obesity^7^. Factors that could contribute to intrapartum complications among women with overweight and obesity include ineffective uterine contractility^38,39^ and fetal macrosomia^7^. Macrosomic infants are at increased risk of a complicated delivery^40^, shoulder dystocia^41^, fetal asphyxia^42^, and subsequent NICU admission^40,43,44^.

The results of this study do not support the hypothesis that ultrasound assessment of VF and SCF in early mid pregnancy could be used as risk marker for neonatal hypoglycemia or adverse neonatal outcomes. This is contrary to the results of a similar study^25^, in which abdominal subcutaneous fat thickness was measured by ultrasound at 18–23.9 weeks’ gestation in 997 women. The fat thickness was associated with neonatal hypoglycemia, low Apgar score, admission to intensive care nursery or special care nursery, and resuscitation^25^. One possible explanation to these contradictive results could be dissimilarities between the study populations. Although the cohorts were similar regarding maternal age, BMI, and time point of subcutaneous fat thickness measurement in relation to conception, other dissimilarities regarding ethnicity, general health of the population, and availability of maternal health care could be present. In addition, the measurement of abdominal subcutaneous fat thickness was assessed closer to the symphysis pubis in their study. This could possibly affect the measure, but should presumably not influence the relation to the outcome.

Our result are also contrary to those reported by Kennedy et al.^26^. Abdominal subcutaneous fat thickness was measured by ultrasound at 11–14 and at 18–24 weeks’ gestation in 1510 women. Associations were found between the first measure and admission to NICU, and between the second measure and neonatal respiratory distress and NICU-admission^26^. Differences between the study populations could possibly explain these contrasting results. For example, the prevalence of obesity was almost twice as high in their cohort (27.0% versus 15.8%), and a larger proportion of their women were parous (63.5% versus 57.2%). Hence, the thickness of the abdominal subcutaneous fat thickness seems to be predictive of adverse neonatal outcomes in a population consisting of more obese women, and more parous women, compared with our study cohort.

Since our results were not consistent with the findings of Eley et al.^25^ and Kennedy et al.^26^, further research is needed before introduction of fat depth measurement can be recommended in clinic. Areas for future research include investigation of the relations between the fat depth measures and other pregnancy outcomes, such as gestational hypertension, preeclampsia, gestational diabetes mellitus, and labor injuries. Moreover, it could be of interest to compare ultrasound measures of VF and SCF with the anthropometry measures waist circumference and waist-to-hip ratio in relation to adverse pregnancy outcomes. The anthropometric measures are cheap and fast to perform, and could for example be added to the assessment of BMI in early pregnancy. In addition, differences in biological pathways between women with a predominance of VF versus women with a predominance of SCF could be a future research area. Even though this study did not find any associations between VF and SCF and adverse neonatal outcomes, we have previously reported on associations between VF and birth size^17^. Visceral fat accumulation in non-pregnant individuals is associated with insulin resistance and inflammation^16^, and to the best of our knowledge, markers of insulin resistance and inflammation in relation to VF and SCF accumulation have not previously been studied in a pregnant population.

The prevalence of neonatal hypoglycemia in our cohort was 2.3%, which is similar to the prevalence of neonatal hypoglycemia reported by a large register based study^45^. Infants identified as at risk (i.e. being preterm, SGA, or having a mother with diabetes) constituted 23.4% of the hypoglycemic infants. Of note, we considered infants that did not undergo blood glucose sampling as normoglycemic. This assumption could be questioned since the normal pattern of blood glucose concentrations after birth are still under investigation. A recent study evaluating the prevalence of hypoglycemia during the first 5 days in a healthy cohort of 67 term infants with no symptoms of hypoglycemia found that 36% of the infants had blood glucose concentrations < 2.6 mmol/l^46^. This finding suggests that seemingly healthy infants with no symptoms of hypoglycemia still might have low blood glucose levels, and that these episodes are undetected.

Interestingly, the sensitivity analyses on a subgroup of healthy women showed an association between SCF and hypoglycemia. This was contrary to our hypothesis that VF, rather than SCF, would be associated with neonatal hypoglycemia. Hence, this finding is in line with the findings of Eley et al.^25^ and Kennedy et al.^26^. It is unclear why this association is present in a cohort of healthy women and not in a cohort including women with chronic illness or pregnancy complications.

The strength of this study was the large sample, consisting of 2771 mother and child-dyads. In Uppsala County, all second-trimester anomaly scans are performed at Uppsala University hospital and more than 97% of all pregnant women are participating^47^, making the study group a population-based cohort. Moreover, the follow up of participants is very good, since Uppsala University Hospital is the only available delivery unit within the county. Of note, the prevalence of NICU admission was lower in our cohort (3.6%) compared with the national admission rate (5.6%) from the same time period^48^, indicating a healthier study population. Although only 2844 out of 12,744 women who had a second-trimester anomaly scan during the study period were undergoing VF and SCF measurements, we believe selection bias was unlikely to take place since it was a coincidence whether the midwife who performed the scan was trained in measuring fat depths or not. Another strength was that neonatal hypoglycemia was identified by the blood glucose concentrations, and not by the ICD-10 codes for neonatal hypoglycemia. In this way, we were able to identify episodes of neonatal hypoglycemia that had been overlooked at the discharge and therefore not registered by an ICD-10 code in the standardized pediatric electronic medical record.

A limitation to the study was that we did not have blood glucose concentrations from all infants in the cohort, and therefore we do not know for sure whether the untested infants had any episodes of hypoglycemia or not. However, since blood specimen collection is painful, it would be unethical to perform blood glucose testing on newborn infants without risk factors and symptoms of hypoglycemia. Thus, the data on blood glucose concentrations we have used in this study are the best available. Another limitation was the high number of missing data on early pregnancy smoking status. Smoking status was therefore imputated to retain power in the statistical analyses. Lastly, the fetal anomaly scans were performed over a wide range of gestational ages. However, the majority of the women had their scan performed in a narrow gestational age range, and there was no correlation between the timing of the measurements in relation to conception and the fat depths. Hence, the timing of the measurements should not affect the overall results of this study.

## Conclusion

The results of this study showed that measurements of VF and SCF by ultrasound in early mid pregnancy were not associated with neonatal hypoglycemia or adverse neonatal outcomes, but confirmed early pregnancy BMI as a risk marker for these outcomes. Further research is needed to determine the usefulness of VF and SCF measures in addition to BMI as markers of adverse pregnancy and neonatal outcomes before any recommendations of implication of fat depth measures in clinical practice can be made.

## Supplementary Information

Supplementary Tables.

## Data Availability

The datasets generated during and/or analyzed during the current study are available from the corresponding author on reasonable request.
