# Supplementary material for: Associations of ultrasound estimated early mid pregnancy visceral and subcutaneous fat depths and early pregnancy BMI with adverse neonatal outcomes
Source: Sci Rep. 2021 Feb 25;11:4612. doi: 10.1038/s41598-021-84045-8 (PMC7907247; doi:10.1038/s41598-021-84045-8)
Supplement: Supplementary file 1 — Supplementary Tables. [file 41598_2021_84045_MOESM1_ESM.pdf]

Supplementary Information file

Associations of ultrasound estimated early mid pregnancy visceral and subcutaneous fat depths and early pregnancy BMI with adverse neonatal outcomes

Emelie Lindberger MD[1*], Anna-Karin Wikström MD PhD[1], Eva Bergman MD PhD[1], Karin Eurenius MD PhD[1], Ajlana Mulic-Lutvica MD PhD[1], Linda Lindström PhD[1], Inger Sundström Poromaa MD PhD[1], Fredrik Ahlsson MD PhD[1]

[1] Department of Women's and Children's Health, Uppsala University, 751 85 Uppsala, Sweden

Supplementary table 1. Overview of outcomes and missing outcome data.

**Outcome**	**n**	**%**	**Mother-child dyads with available data in the cohort n =2771, n (%)**
**Composite outcome[a]**			2077 (75.0)
Yes	137	6.6	
No	1940	93.4	
**Apgar score <7 at 5 minutes of age**			2635 (95.1)
Yes	42	1.6	
No	2593	98.4	
**Umbilical artery pH ≤7.0**			2118 (76.4)
Yes	19	0.9	
No	2099	99.1	
**Admission to NICU**			2771 (100.0)
Yes	99	3.6	
No	2672	96.4	

[a]Apgar <7 at 5 minutes of age, or umbilical artery pH ≤7.0, or admission to NICU
NICU, neonatal intensive care unit

Supplementary table 2. Pearson correlation coefficients for the linear associations between early pregnancy BMI, maternal age, visceral fat depth and subcutaneous fat depth.

	**Early pregnancy BMI**	**Age**	**Visceral fat**	**Subcutaneous fat**
**Early pregnancy BMI**		0.03	0.49**	0.67**
**Age**			0.08**	-0.01
**Visceral fat**				0.32**

** *P* <0.01
BMI, body mass index

Supplementary table 3. Categorical covariates in relation to outcomes.

	Variable	Neonatal hypoglycemia		*P*-value	Composite outcome[a]		*P*-value	Apgar <7 at 5 minutes of age		*P*-value	Umbilical artery pH ≤7.0		*P*-value	Admission to NICU		*P*-value
		Yes n =63	No n =2687		Yes n =126	No n =1936		Yes n =41	No n =2579		Yes n =19	No n =2088		Yes n =88	No n =2662	
**Women**	Nulliparous (n)	34	1151	0.090[b]	64	838	0.422[b]	22	1107	0.208[b]	9	910	0.725[b]	46	1139	0.449[b]
	Parous (n)	30	1556		73	1102		20	1486		10	1189		53	1533	
	Non-smoker at first antenatal visit (n)	62	2610	1.000[c]	133	1863	0.540[b]	41	2498	1.000[c]	19	2018	1.000[c]	96	2576	1.000[c]
	Smoker at first antenatal visit (n)	2	97		4	77		1	95		0	81		3	96	
	Born in EU (n)	57	2342	0.555[b]	117	1687	0.602[b]	35	2246	0.536[b]	19	1825	0.159[c]	83	2316	0.416[b]
	Born outside EU (n)	7	365		20	253		7	347		0	274		16	356	

[a]Apgar <7 at 5 minutes of age, or umbilical artery pH ≤7.0, or admission to NICU
NICU, neonatal intensive care unit

[b]*P*-values are for Chi square tests

[c]*P*-values are for Fisher's exact tests

Supplementary table 4. Maternal age in association with outcomes.

	Neonatal hypoglycemia		*P*-value[a]	Composite outcome[b]		*P*-value[a]	Apgar <7 at 5 minutes of age		*P*-value[a]	Umbilical artery pH ≤7.0		*P*-value[a]	Admission to NICU		*P*-value[a]
	**Yes**	**No**		**Yes**	**No**		**Yes**	**No**		**Yes**	**No**		**Yes**	**No**	
**n**	64	2707	0.163	137	1940	0.477	**42**	**2593**	**0.022**	19	2099	0.555	99	2672	0.780
**Maternal age, years (mean ± SD)**	31.2 (5.7)	30.3 (4.8)		30.6 (5.2)	30.3 (4.8)		**32.0 (4.4)**	**30.3 (4.8)**		31.0 (5.6)	30.3 (4.8)		30.2 (5.3)	30.3 (4.8)	

Data are means ± standard deviations.
[a]*P*-values are for independent t-tests.
[b]Apgar <7 at 5 minutes of age, or umbilical artery pH ≤7.0, or admission to NICU; NICU, neonatal intensive care unit

Supplementary table 5. Associations between early mid pregnancy visceral fat depth, subcutaneous fat depth, early pregnancy BMI, and outcomes in a subgroup of healthy women (n =2496).

	**Visceral fat depth**						**Subcutaneous fat depth**						**BMI**					
**Outcome**	**Unadjusted model**			**Adjusted model[b]**			**Unadjusted model**			**Adjusted model[b]**			**Unadjusted model**			**Adjusted model[c]**		
	OR	CI	*P*	OR	CI	*P*	OR	CI	*P*	OR	CI	*P*	OR	CI	*P*	OR	CI	*P*
**Neonatal hypoglycemia**	1.09	1.00–1.19	0.063	1.03	0.93–1.14	0.535	**1.34**	**1.14–1.59**	**<0.001**	**1.27**	**1.01–1.60**	**0.038**	**1.07**	**1.02–1.13**	**0.007**	**1.08**	**1.02–1.14**	**0.005**
**Composite outcome[a]**	1.00	0.94–1.06	0.980	0.96	0.90–1.03	0.279	1.08	0.95–1.22	0.230	0.96	0.81–1.14	0.616	**1.04**	**1.01–1.08**	**0.025**	**1.05**	**1.01–1.08**	**0.020**
**Apgar <7 at 5 minutes of age**	1.01	0.90–1.11	0.989	0.96	0.86–1.08	0.517	1.09	0.89–1.34	0.398	1.01	0.76–1.34	0.948	1.03	0.97–1.10	0.341	1.04	0.97–1.10	0.280
**Umbilical artery pH ≤7.0**	0.96	0.83–1.11	0.540	0.94	0.80–1.11	0.461	0.89	0.64–1.24	0.489	0.75	0.48–1.16	0.195	1.02	0.93–1.11	0.742	1.02	0.93–1-12	0.710
**Admission to NICU**	1.03	0.96–1.11	0.426	0.99	0.91–1.08	0.808	**1.19**	**1.03–1.37**	**0.016**	1.10	0.90–1.35	0.334	**1.05**	**1.01– 1.10**	**0.018**	**1.06**	**1.01–1.10**	**0.016**

Data are odds ratios (OR) (95% confidence interval (CI)) for the change in outcome per five mm increase in fat depth and per unit increase in BMI (kg/m^2).

Data were analyzed using logistic regression models.

[a]Apgar <7 at 5 minutes of age, or umbilical artery pH ≤7.0, or admission to NICU

[b]Adjustments in the model: early pregnancy BMI, age, smoking at first antenatal visit, parity, and country of birth.

[c]Adjustments in the model: maternal age, smoking at first antenatal visit, parity, and country of birth.

NICU, neonatal intensive care unit; BMI, body mass index
